# A rare case of persistent sciatic artery associated with arteriosclerotic occlusive disease

**DOI:** 10.1093/jscr/rjaf384

**Published:** 2026-05-16

**Authors:** Tomohiro Nakajima, Tsuyoshi Shibata, Yutaka Iba, Keishi Ogura, Nobuyoshi Kawaharada

**Affiliations:** Department of Cardiovascular Surgery, Sapporo Medical University School of Medicine, Sapporo, Japan; Department of Cardiovascular Surgery, Sapporo Medical University School of Medicine, Sapporo, Japan; Department of Cardiovascular Surgery, Sapporo Medical University School of Medicine, Sapporo, Japan; Division of Radiology and Nuclear Medicine, Sapporo Medical University Hospital, Sapporo, Japan; Department of Cardiovascular Surgery, Sapporo Medical University School of Medicine, Sapporo, Japan

**Keywords:** arteriosclerosis obliterans, residual sciatic artery, endovascular therapy, arterioplasty, patent ductus arteriosus

## Abstract

To present a rare case of persistent sciatic artery (PSA) complicated by arteriosclerotic occlusive disease and discuss the diagnostic and therapeutic challenges. Methods: Retrospective analysis of clinical findings, imaging results, and surgical interventions in a 61-year-old female presenting with left leg numbness and intermittent claudication. Imaging revealed PSA with concurrent stenosis of the external iliac and femoral arteries. Treatment involved endovascular stenting followed by open surgical intervention, resulting in significant symptom improvement. This case highlights the importance of a multidisciplinary approach in managing rare vascular anomalies, integrating endovascular and open surgical techniques to achieve optimal outcomes.

## Introduction

Persistent sciatic artery (PSA) is a rare vascular anomaly resulting from incomplete regression of the embryological sciatic artery, which typically regresses as the femoral artery develops into the dominant blood supply to the lower extremity [1]. This congenital condition has an incidence of 0.025%–0.04% and is often asymptomatic, discovered incidentally during imaging or in association with complications, such as aneurysm formation, thrombosis, or embolism [2, 3].

PSA presents significant clinical challenges, particularly when it is associated with arteriosclerotic changes or complex vascular pathologies. The management of PSA, especially when complicated by aneurysmal degeneration, requires careful planning to avoid limb ischemia or embolic events, while preserving arterial flow to the lower limb [4].

## Case report

The patient was a 61-year-old female who underwent cannulation of the left femoral artery during childhood for surgical repair of an atrial septal defect. No follow-up was conducted thereafter, leaving the details of her medical history unclear. At the age of 57, she presented to another hospital with intermittent claudication. Diagnostic evaluation revealed significant stenosis of the left external iliac artery, for which endovascular treatment was performed.

**Figure 1:**
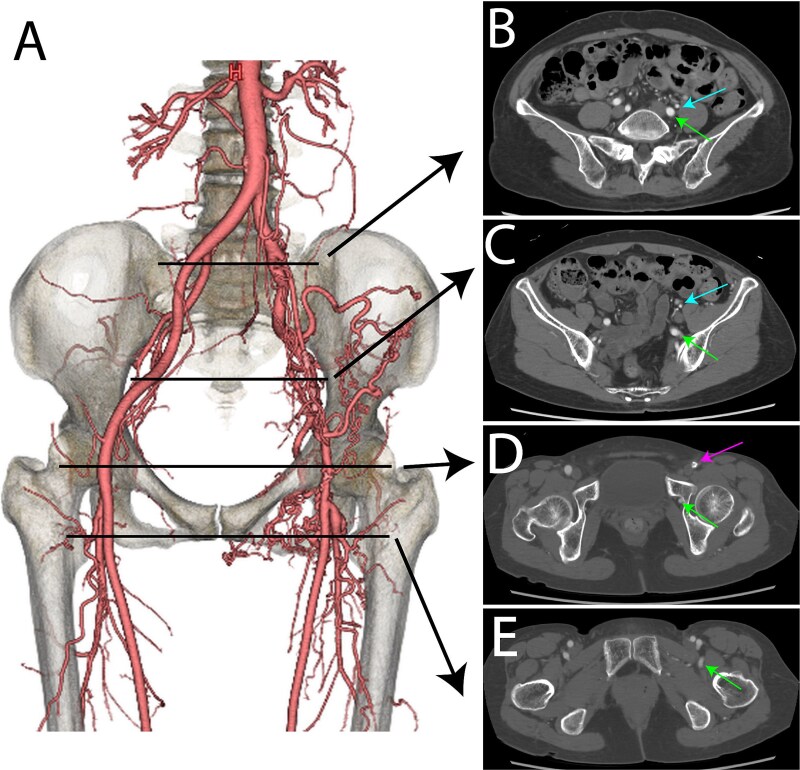
Preoperative contrast-enhanced CT images. (A) Volume-rendered (VR) image. (B) Bifurcation level of the external iliac artery (blue arrow) and the internal iliac artery. The sciatic artery is marked with a green arrow. (C) Pelvic level. The external iliac artery (blue arrow) and the sciatic artery (green arrow) are shown. (D) Femoral head level. The left common femoral artery (pink arrow) and the sciatic artery (green arrow) are displayed. (E) Postbifurcation of the superficial femoral artery and deep femoral artery. The sciatic artery (green arrow) is visible.

At 61, she presented to our institution with numbness in her left leg during ambulation. Ankle-brachial indices (ABIs) were 1.21 on the right and 0.95 on the left, within the normal range for the left. Contrast-enhanced computed tomography (CT) revealed stenosis of the left external iliac artery, severe stenosis of the left common femoral artery, and a remnant sciatic artery (Fig. 1). Based on Pillet’s classification, the condition was classified as type 3.

**Figure 2:**
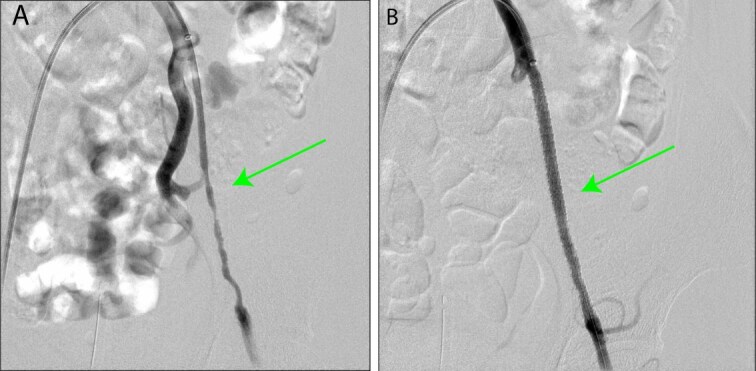
Intraoperative images taken during endovascular treatment. (A) Preangiography image of the left external iliac artery (green arrow). (B) Postangiography image of the left external iliac artery (green arrow). A 7 × 58 mm LifeStream™ balloon-expandable covered stent (Bard Peripheral Vascular, Inc., Tempe, AZ, USA) was placed.

Given the stenosis of the external iliac artery, the patient underwent endovascular treatment. A LIFE STREAM™ balloon-expandable covered stent (7 × 58 mm; Bard Peripheral Vascular, Inc., Tempe, AZ, USA) was deployed in the left external iliac artery (Fig. 2). Although post-treatment follow-up revealed persistent numbness during exercise, the patient continued to be monitored as an outpatient. Three months later, following discussions with the patient, surgical intervention was planned to address the stenosis of the left common femoral artery.

**Figure 3:**
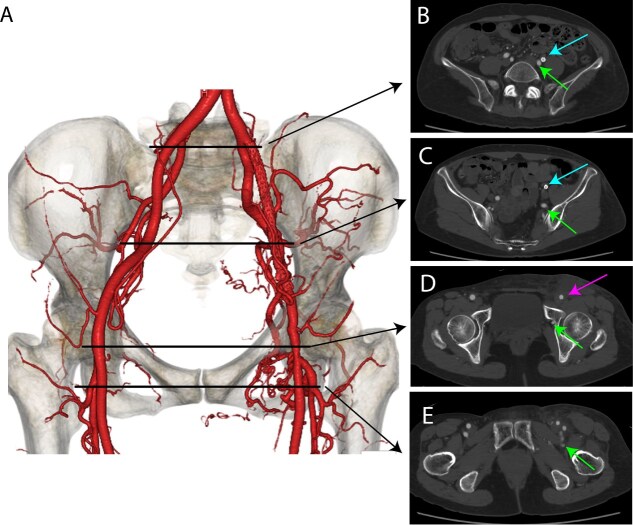
Postoperative contrast-enhanced CT images. (A) VR image. (B) Bifurcation level of the external iliac artery (blue arrow) and the internal iliac artery. The sciatic artery is marked with a green arrow. Improved blood flow is observed in the left external iliac artery. (C) Pelvic level. The external iliac artery (blue arrow) and the sciatic artery (green arrow) are shown. Improved blood flow is observed in the left external iliac artery. (D) Femoral head level. The left common femoral artery (pink arrow) and the sciatic artery (green arrow) are displayed. The lumen of the left common femoral artery appears widened. (E) Postbifurcation of the superficial femoral artery and deep femoral artery. The sciatic artery (green arrow) is visible.

Under general anesthesia, a vertical incision was made to expose the left femoral artery. Endarterectomy was performed on the left common femoral artery, followed by angioplasty using a XenoSure® Biologic Vascular Patch (LeMaitre Vascular, Inc., Burlington, MA, USA) (Fig. 3). Post-operatively, the patient experienced mild improvement in her symptoms of exercise-induced numbness.

## Discussion

This case highlights the complexity of diagnosing and managing vascular anomalies in the context of prior surgical intervention. Although the observed vascular pattern was consistent with a PSA, alternative explanations should be considered [5]. One possibility is that the patient’s history of femoral artery cannulation during childhood may have damaged the left external iliac or femoral arteries, resulting in stenosis [6]. Over time, compensatory hypertrophy and remodeling of the internal iliac artery may have occurred, creating a vascular pathway resembling that of the PSA. This raises the intriguing question of whether the anomaly represents a true congenital persistence of the sciatic artery or a secondary adaptation to a previous iatrogenic injury. This distinction is clinically significant as it informs both diagnostic considerations and surgical planning in similar cases.

The persistence of exercise-induced paresthesia in the left lower limb, despite successful surgical intervention, warrants further investigation. Preoperatively, the patient’s ABI remained above 0.90, suggesting adequate baseline perfusion [7]. Postoperative imaging confirmed the restoration of blood flow, effectively addressing the ischemic component. However, continued paresthesia raises the possibility of a nonvascular etiology, such as neuropathy, muscular strain, or other functional factors unrelated to ischemia. This underscores the importance of preoperative counseling in managing patient expectations and the need for a multidisciplinary approach when evaluating persistent symptoms. Further investigations should include electromyography or nerve conduction studies to explore potential neurological contributors.

Another consideration is the timing and decision-making regarding surgical intervention. Given that the patient had a functional ABI preoperatively, the indications for surgery were primarily driven by symptom relief and quality-of-life concerns. This highlights the challenge in balancing the risks and benefits of invasive procedures in patients with complex vascular anomalies. Despite the successful restoration of blood flow, the patient’s residual symptoms underscore the need for comprehensive preoperative evaluations, including the potential role of advanced imaging and functional studies, to fully understand the interplay between vascular and non-vascular contributors to symptoms [8]. This case demonstrates the complexities associated with diagnosing and managing PSA with concurrent arteriosclerotic disease. The integration of endovascular and surgical interventions allowed for effective symptom resolution, highlighting the importance of individualized treatment strategies in managing rare vascular anomalies.
